# Reemerging Sudan Ebola Virus Disease in Uganda, 2011

**DOI:** 10.3201/eid1809.111536

**Published:** 2012-09

**Authors:** Trevor Shoemaker, Adam MacNeil, Stephen Balinandi, Shelley Campbell, Joseph Francis Wamala, Laura K. McMullan, Robert Downing, Julius Lutwama, Edward Mbidde, Ute Ströher, Pierre E. Rollin, Stuart T. Nichol

**Affiliations:** US Centers for Disease Control and Prevention, Entebbe, Uganda (T. Shoemaker, S. Balinandi, R. Downing);; Centers for Disease Control and Prevention, Atlanta, Georgia, USA (A. MacNeil, S. Campbell, L.K. McMullan, U. Ströher, P.E. Rollin, S.T. Nichol);; Ministry of Health, Kampala, Uganda (J.F. Wamala);; and Uganda Virus Research Institute, Entebbe (J. Lutwama, E. Mbidde)

**Keywords:** viral hemorrhagic fever, VHF, Ebola, Ebola hemorrhagic fever, EHF, outbreak, *Sudan ebolavirus*, Sudan Ebola virus disease, Ebola virus, Ebolavirus, Filovirus, epidemiology, viruses, EBOV, SEBOV, Uganda, reemerging

## Abstract

Two large outbreaks of Ebola hemorrhagic fever occurred in Uganda in 2000 and 2007. In May 2011, we identified a single case of Sudan Ebola virus disease in Luwero District. The establishment of a permanent in-country laboratory and cooperation between international public health entities facilitated rapid outbreak response and control activities.

## The Patient

On May 6, 2011, a 12-year-old girl from Nakisamata village, Luwero District, Uganda, was admitted to Bombo Military Hospital. She exhibited fever, jaundice, and hemorrhagic signs: epistaxis, hematemesis, hematuria, and conjunctival, gingival, and vaginal bleeding. The attending physician made a preliminary diagnosis of disseminated intravascular coagulopathy with a functional platelet disorder, along with viral hemorrhagic fever (VHF) as a possible cause. The patient was isolated from the general ward because of a high clinical suspicion of VHF. Hospital staff involved in her care and treatment implemented isolation precautions, including the use of personal protective equipment, such as gowns, gloves, and masks.

The patient’s condition worsened and despite tracheal intubation and supplemental oxygen, she died 3 hours after admission. Because the cause of death was unknown but suspicion of VHF was high, the body was disinfected by using a chlorine solution and then wrapped in plastic, taken to the hospital mortuary facility, and placed in a coffin, which was then sealed. The coffin was released to the girl’s relatives for burial, with instructions not to open the coffin or touch the body before burial.

A blood sample collected at the hospital before the patient’s death was transported to the US Centers for Disease Control/Uganda Virus Research Institute (CDC/UVRI) laboratory in Entebbe for diagnostic testing by reverse transcription PCR (RT-PCR), antigen-detection ELISA, and IgM for filoviruses as described (*1*–*5*). Evidence of infection with an Ebola virus of the genus and species *Ebolavirus* *Sudan ebolavirus* (SEBOV) was detected by RT-PCR and confirmed by antigen-detection ELISA. Results of ELISA IgM against Ebola viruses and all tests for Marburg virus were negative. SEBOV was also isolated from blood on Vero E6 cells at the Viral Special Pathogens Branch, CDC, Atlanta, GA, USA.

**Figure 1:**
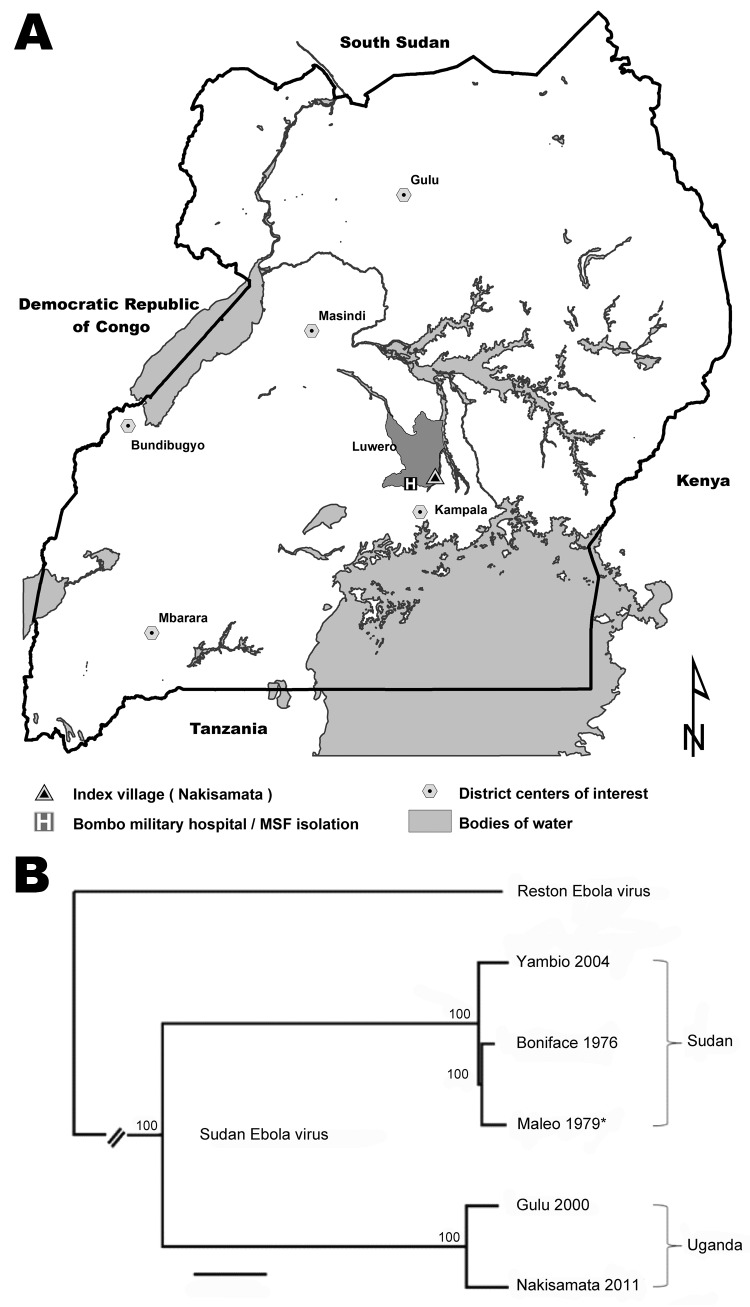
Sudan Ebola virus in Uganda, 2011. A) Geographic locations of Nakisimata village and Bombo Military Hospital with the isolation facility established by Médecins Sans Frontières (MSF) relative to locations where Sudan Ebola virus (SEBOV) was isolated during the current and previous outbreaks in Uganda. Scale bar indicates kilometers. B) Maximum likelihood tree obtained from full length sequences of SEBOV strains Nakisamata (JN638998), Boniface (FJ968794), Gulu (AY729654), and Yambio (EU338380) and the polymerase gene (*) of Maleo (U23458); full-length Reston Ebola virus (REBOV) (AY769362) is included as an outgroup. Bootstrap values listed at the nodes provide statistical support for 1,000 replicates. Scale bar indicates 0.006 substitutions per site.

Overlapping PCR fragment copies of the complete virus genome were amplified, and the nucleotide sequence was obtained as described (*6*). Maximum-likelihood phylogenetic analysis confirmed SEBOV and demonstrated that the isolate (Nakisamata isolate, JN638998) was closely related (99.3% identical) to the Gulu SEBOV strain obtained from northern Uganda in 2000 (Figure 1). A postmortem diagnosis indicated Ebola hemorrhagic fever (EHF) caused by SEBOV as the cause of the patient’s death.

An investigation team from the Uganda Ministry of Health, CDC Uganda, and UVRI traveled to Bombo Military Hospital and Nakisamata village, the home of the case-patient, on May 13, 2011. The village is located in Luwero District, ≈50 km north of Kampala. The investigation team established that the case-patient reported feeling ill on May 1. She had a mild headache and was given an over-the-counter analgesic. She had a fever with chills on May 4 and began vomiting on May 5. On May 6, she experienced intense fatigue and epistaxis. The patient’s grandmother then took her to a local health clinic where she received adrenaline nasal packs for her epistaxis and injections of quinine and vitamin K. The patient’s condition continued to worsen, and she experienced hematemesis and vaginal bleeding. She was then transported by motorcycle taxi to Bombo Military Hospital, ≈35 km north of Kampala, by her grandmother and father.

The investigation team identified 25 close contacts of the patient, comprising 13 persons who had physical contact after illness onset at her home and 12 hospital staff members. Four of the hospital contacts were classified as having a high risk for exposure to SEBOV because of possible exposure to the patient’s body fluids: 2 persons who performed tracheal intubation and 2 who handled the body after death. On May 15, a team from CDC Atlanta arrived in Uganda to provide additional assistance in laboratory diagnostics and epidemiological response. Also on this date, Médecins Sans Frontières, a nonprofit medical humanitarian organization, began establishment of an isolation ward at Bombo Military Hospital. During outbreak response and follow-up surveillance 21 days after the death of the case-patient, 24 sick persons (18 from Luwero District and 6 from other locations in Uganda) were identified. Testing at the CDC/UVRI laboratory ruled out EHF in this group.

Relatives reported that the girl did not travel outside Nakisamata village in the 3 months preceding her illness and did not attend any funerals or have contact with anyone visiting from another town or village before her illness. They recalled no unusual deaths in the area in recent months. They also reported that she had not been exposed to any sick or dead animals in the village or nearby forested area.

During follow-up investigation in Nakisamata village, several species of bats (tentatively identified as belonging to the genera *Epomophorus*, *Hipposideros*, *Pipistrellus*, and *Chaerophon*) were found roosting in unoccupied houses and several classrooms of the village schoolhouse where the girl attended classes, ≈400 meters from her home. Sixty-four bats were collected; testing of these bats found no evidence of Ebola virus (EBOV) infection, but ecological studies in Uganda are ongoing.

Samples from 4 family members, none of whom reported illness, were obtained and tested for EBOV by RT-PCR, antigen-detection ELISA, and IgM and IgG ELISAs. Test results for 3 of the family members were negative. One juvenile relative had positive IgG test results at a titer of 1,600 but was IgM negative, indicating past infection with EBOV. Since IgM antibodies can persist for as long as 2 months after infection (*1*,*7*), this person’s infection appears temporally unrelated to the case-patient, who had EHF attributed to SEBOV. No clinical information was available to determine whether the relative’s infection was symptomatic. Contact studies and serosurveys suggest that some EBOV infections can go unrecognized (*1*,*8*–*10*,*11*).

## Conclusions

This case represents the second documented occurrence of an identified single-case EHF outbreak (*12*). We were unable to identify an epidemiologic link to any suspected EHF cases before the girl’s illness onset, or to conclusively identify a suspected environmental source of infection in and around the village in which she lived. This suggests that her exposure was zoonotic in nature and must have occurred in the vicinity of her residence, since her relatives reported that she did not travel. The fact that an additional family member had serologic evidence of an epidemiologically unrelated EBOV infection further supports the notion that zoonotic exposures have occurred in the vicinity of the case-patient’s village.

Rapid laboratory identification in this outbreak supported mobilization of an investigation team 1 day after initial laboratory detection and the rapid establishment of an isolation facility at Bombo Military Hospital. In this instance, the initial high suspicion of EHF by clinical staff, the appropriate use of personal protective equipment and barrier protection by hospital staff, and the rapid laboratory confirmation of EHF in-country likely contributed to limiting the size of this outbreak.

**Figure 2:**
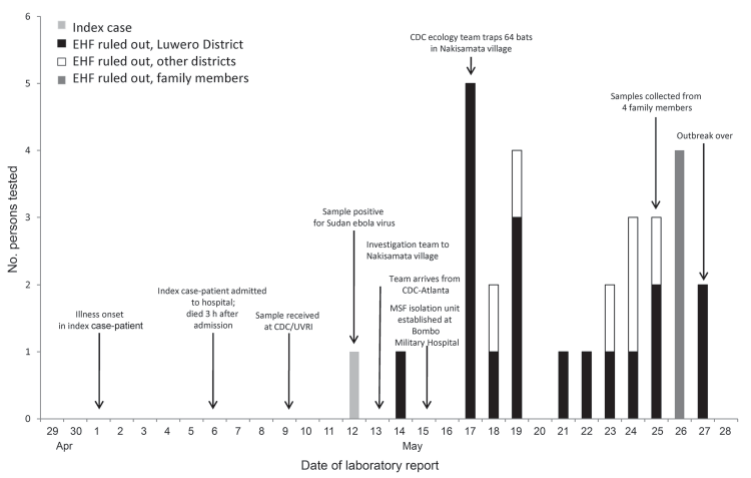
Timeline of Sudan Ebola virus outbreak, Uganda 2011, showing key events in the investigation and response. Also shown are the dates on which EHF was ruled out in other suspected cases by laboratory testing at the CDC/UVRI laboratory in Entebbe. EHF, Ebola hemorrhagic fever; CDC/UVRI, US Centers for Disease Control and Prevention, Uganda/Uganda Virus Research Institute Collaborative Laboratory; CDC, US Centers for Disease Control and Prevention; MSF, Médecins Sans Frontières.

The timeliness of diagnostic confirmation and outbreak response was much improved over that during previous EHF outbreaks in Uganda (timeline shown in Figure 2), during which transmission of the virus occurred for multiple months before the outbreaks were detected (*13*–*15*). This improvement was possible mainly because of collaboration by the CDC Viral Special Pathogens Branch and the Uganda Virus Research Institute to establish a permanent high-containment laboratory that is capable of performing diagnostic testing for filoviruses and other causes of VHF in Uganda. The limited extent of this outbreak also demonstrates the powerful utility of a national VHF surveillance system, coupled with the ability to rapidly diagnose and respond to limit the spread of such high-hazard infections in the community and health care facilities. Continued efforts are needed to build and sustain VHF surveillance networks across Africa.
